# Gait Kinematic and Kinetic Characteristics of Older Adults With Mild Cognitive Impairment and Subjective Cognitive Decline: A Cross-Sectional Study

**DOI:** 10.3389/fnagi.2021.664558

**Published:** 2021-08-03

**Authors:** Qian Zhong, Nawab Ali, Yaxin Gao, Han Wu, Xixi Wu, Cuiyun Sun, Jinhui Ma, Lehana Thabane, Ming Xiao, Qiumin Zhou, Ying Shen, Tong Wang, Yi Zhu

**Affiliations:** ^1^Rehabilitation Medicine Center, The First Affiliated Hospital of Nanjing Medical University, Nanjing, China; ^2^Department of Rehabilitation, Nanjing Drum Tower Hospital Clinical College of Nanjing Medical University, Nanjing, China; ^3^Swat Institute of Rehabilitation & Medical Sciences, Swat, Pakistan; ^4^Department of Rehabilitation, The Affiliated Suzhou Hospital of Nanjing Medical University, Suzhou Municipal Hospital, Gusu School, Nanjing Medical University, Suzhou, China; ^5^Zhongshan Rehabilitation Branch, The First Affiliated Hospital of Nanjing Medical University, Nanjing, China; ^6^Department of Health Research Methods, Evidence, and Impact, McMaster University, Hamilton, ON, Canada; ^7^Biostatistics Unit, St. Joseph’s Healthcare, Hamilton, ON, Canada; ^8^Jiangsu Key Laboratory of Neurodegeneration, Center for Global Health, Nanjing Medical University, Nanjing, China; ^9^Brain Institute, The Affiliated Nanjing Brain Hospital of Nanjing Medical University, Nanjing, China

**Keywords:** mild cognitive impairment, subjective cognitive decline, gait, kinematics, kinetics

## Abstract

**Background:**

Older adults with mild cognitive impairment (MCI) have slower gait speed and poor gait performance under dual-task conditions. However, gait kinematic and kinetic characteristics in older adults with MCI or subjective cognitive decline (SCD) remain unknown. This study was designed to explore the difference in gait kinematics and kinetics during level walking among older people with MCI, SCD, and normal cognition (NC).

**Methods:**

This cross-sectional study recruited 181 participants from July to December 2019; only 82 met the inclusion criteria and consented to participate and only 79 completed gait analysis. Kinematic and kinetic data were obtained using three-dimensional motion capture system during level walking, and joint movements of the lower limbs in the sagittal plane were analyzed by Visual 3D software. Differences in gait kinematics and kinetics among the groups were analyzed using multivariate analysis of covariance (MANCOVA) with Bonferroni *post-hoc* analysis. After adjusting for multiple comparisons, the significance level was *p* < 0.002 for MANCOVA and *p* < 0.0008 for *post-hoc* analysis.

**Results:**

Twenty-two participants were MCI [mean ± standard deviation (SD) age, 71.23 ± 6.65 years], 33 were SCD (age, 72.73 ± 5.25 years), and 24 were NC (age, 71.96 ± 5.30 years). MANCOVA adjusted for age, gender, body mass index (BMI), gait speed, years of education, diabetes mellitus, and Geriatric Depression Scale (GDS) revealed a significant multivariate effect of group in knee peak extension angle (*F* = 8.77, *p* < 0.0001) and knee heel strike angle (*F* = 8.07, *p* = 0.001) on the right side. *Post-hoc* comparisons with Bonferroni correction showed a significant increase of 5.91° in knee peak extension angle (*p* < 0.0001) and a noticeable decrease of 6.21°in knee heel strike angle (*p* = 0.001) in MCI compared with NC on the right side. However, no significant intergroup difference was found in gait kinetics, including dorsiflexion, plantar flexion, knee flexion, knee extension, hip flexion, and hip extension(*p* > 0.002).

**Conclusion:**

An increase of right knee peak extension angle and a decrease of right knee heel strike angle during level walking were found among older adults with MCI compared to those with NC.

## Introduction

Gait disturbance and cognitive decline increase with advancing age (Cohen et al., 2016), and both of these are considered prominent risk factors of fall in older people with dementia (Ambrose et al., 2013; Rinaldi and Moraes, 2016; Zhang et al., 2019). Therefore, the investigation of gait characteristics and its correlation with cognition is of great importance for fall prevention and cognitive improvement in older individuals with cognitive decline.

In a recent urban community cohort study, gait abnormality was diagnosed by clinicians in 35% of older adults (Verghese et al., 2006). In general, the older population shows slower preferred walking speed, reduced cadence, shorter step and stride length, and a cautious gait; however, gait variability remains stable over time (Herssens et al., 2018). Cognitive impairment is considered one of the risk factors associated with slow gait velocity, physical inactivity, muscle weakness, pain, impaired vision, prior history of falls, and obesity in older individuals (Verghese et al., 2016). Furthermore, slow gait velocity (slowing of gait) is one of the early signs of dementia; compared with healthy seniors, it further declines with progress of the severity of the disease (Kikkert et al., 2016). Higher stride time variability, longer Timed Up & Go test delta time, and slower gait speed are associated with a decline in episodic memory and executive performances in community-dwelling elderly without dementia (Beauchet et al., 2014). Quantitative tests have revealed gait dysfunction in subjects with amnestic and non-amnestic mild cognitive impairment (MCI) subtypes compared with healthy controls (Verghese et al., 2008). Furthermore, cognitive impairment has been found to affect spatiotemporal parameters of gait under dual-task performance (Montero-Odasso et al., 2014; Muurling et al., 2020).

Gait kinematics is the study of joint angles and segment orientation during walking. There is a wide variety of studies in the literature about the joint angle and range of motion (ROM) of the ankle, knee, and hip in the sagittal plane. Previous studies have shown that aged individuals have a reduced ankle ROM and ankle plantar flexion angle, increased knee ROM, greater hip flexion at heel strike and peak hip flexion, and less hip extension with increased hip ROM compared with young adults during walking. However, when walking speeds are matched, the difference in knee kinematics becomes more prominent (Boyer et al., 2017). Reduced strength and passive ROM of the lower extremity contribute to gait disturbances with advancing age (Kang and Dingwell, 2008; Ko et al., 2012), further leading to less upright posture during walking. Aged individuals also need greater joint effort at the hip and knee than at the ankle joints (DeVita and Hortobagyi, 2000), which might represent biomechanical plasticity (Anderson and Madigan, 2014; Kuhman et al., 2018). Furthermore, neuromuscular control of gait adaptation to age-related physiological changes and intersegmental coordination, especially foot–shank coordination, has been observed in the aged population (Hortobágyi et al., 2016; Gueugnon et al., 2019). Some studies have shown that the decline in gait control might lead to typical kinematic and kinetic gait changes among older adults. Although gait kinematics and kinetics have been studied well in the aged population, it remains unclear whether cognitive decline influences these gait parameters. Furthermore, detailed gait kinematics and kinetics in patients with MCI and subjective cognitive decline (SCD) have not been reported so far. Therefore, the aim of the present study was to analyze gait kinematic and kinetic characteristics in older adults with MCI and SCD and to further explore the relationship between gait and cognition.

## Materials and Methods

### Participants

Older individuals from the local community were recruited if they satisfied the following criteria: (1) age between 55 and 85 years; (2) no history of stroke, cerebral hemorrhage, brain tumor, head trauma, or Parkinson’s disease; (3) no walking disability, severe arthritis, diabetic foot, fracture of the lower limb, or other related conditions; (4) junior high school education or above; (5) no unstable cardiovascular disease, no liver, and renal function failure. The participants were screened by neuropsychologists at the memory clinic of the First Affiliated Hospital of Nanjing Medical University between July and December 2019.

### Inclusion and Exclusion Criteria

Screened individuals were recruited for this cross-sectional study if they met the inclusion criteria for MCI, SCD, or normal cognition (NC), and if they provided written consent.

While screening the medical history, a neurologist assessed Clinical Dementia Rating (CDR) (Lam et al., 2008), Mini-Mental State Examination (MMSE) (Folstein et al., 1975), and Hachinski Ischemic Score (HIS) (Swanwick et al., 1996) to exclude patients with dementia and vascular dementia.

In addition, participants were excluded if they had at least one of the following exclusion criteria: (1) diagnosis of vascular dementia, with HIS (Swanwick et al., 1996) of more than 4; (2) CDR score at least 1.0; (3) MMSE < 24; (4) presence of structural abnormality that could affect cognitive function, including brain tumor, subdural hematoma, previous head trauma, neurologic, or psychiatric disease; (5) had medical intervention that could impair cognitive function or treated for depression, unable to take part in cognitive function tests and gait analysis; (6) presence of deformities that affect walking; and (7) disorder such as deafness, blindness, severe language disorder, or physical disability.

Afterward, the participants were assessed using the following neuropsychological tests, covering three cognitive domains: (1) *episodic memory* assessed by Auditory–Verbal Learning Test–Huashan version (AVLT-H) (Zhao et al., 2012), using the delayed recall and delayed recognition scores; (2) *speed/executive function* assessed by trail-making test (TMT) parts A and B (Perrochon and Kemoun, 2014), using the time spent for completing TMT A and TMT B; and (3) *language function* assessed by verbal fluency (McDonnell et al., 2020) and Boston Naming Test (Stålhammar et al., 2016), using the scores of both tests. Meanwhile, depression level was assessed using the 30-item Geriatric Depression Scale (GDS-30) (Chau et al., 2006).

The inclusion criteria for MCI were based on the results of the abovementioned neuropsychological tests (Bondi et al., 2014) and the recommendations for MCI diagnosis in China (Han, 2018) along with memory complaint for more than 6 months. The participants were considered to have MCI if they met at least one of the following criteria: (1) impaired score, defined as >1 standard deviation (SD) below the age-corrected normative means, on both scores for at least one cognitive domain (memory, speed/executive function, or language); (2) one impaired score, defined as >1 SD below the age-corrected normative mean, in each of the three cognitive domains (memory, speed/executive function, or language). The normative means used in this study were taken from Guo et al. as used in Chinese population studies (Zhao et al., 2012) and summarized by Li et al. (2019).

Self-reported questionnaires (Blom et al., 2019) in line with the suggestions of SCD Initiative Working Group (Jessen et al., 2014) were used to discriminate SCD from NC. The inclusion criteria for SCD were as follows: (1) self-experienced persistent decline in memory rather than other domains of cognition for more than 6 months; (2) concerns related to SCD and feeling of deteriorating performance compared to the same age-group individuals; and (3) performance on standardized cognitive tests with age, gender, and education-adjusted norms, i.e., without meeting the diagnostic criteria for MCI or dementia.

The inclusion criteria for NC group were as follows: (1) not fulfilling the diagnosis of SCD or MCI; and (2) no complaints of cognitive impairment or memory loss.

This study was approved by the Ethics Committee of the First Affiliated Hospital of Nanjing Medical University (also named Jiangsu Province Hospital) (Approval Number: 2019-SR-015). All of the participants signed a written consent.

### Gait Analysis

All the participants completed the gait analysis in the gait lab at Zhongshan Rehabilitation Branch of The First Affiliated Hospital of Nanjing Medical University. Motion capture system (Vicon Nexus 2.8, Oxford Metrics, Oxford, United Kingdom) with 12 cameras (Vantage5, Vicon Nexus 2.8, Oxford Metrics, Oxford, United Kingdom) was used to capture movement data of the markers put on the main joints (based on a 51-marker model) of these individuals. The model named Conventional Gait Model 2 (CGM 2.3 vision), which was developed by Dr. Fabien Leboeuf (University of Salford) and partly funded by Vicon, was used. It is an open-source biomechanical model developed in Python 2. The markers were attached to various parts of the body [in front and behind the head, acromion, supraclavicular fossa, manubrium, lateral elbow, medial and lateral wrist, hand, in the middle of the second and third metacarpophalangeal joints, seventh cervical vertebra, 10th thoracic vertebra, anterior and posterior superior iliac spine, medial and lateral knee, medial and lateral ankle, heels, and toes (in the middle of the second and third metatarsals)], while the tracking markers were set at the middle of the upper limb and forearm, right side of the scapula, proximal anterior thigh, distal anterior and lateral thigh, proximal and middle anterior crest of the tibia, lateral epicondyle, and medial malleolus. Kinetic data were recorded by two force plates (ATMI BP400600, United States, sampling at 1,000 Hz), embedded in the floor of the 10-m walkway path. Thereafter, the subjects were instructed to walk 10 m five times at their usual speed. The time it took to walk the middle 6 m was measured, and the ankle, knee, and hip kinematics in the sagittal plane were measured as primary outcomes.

### Kinematic and Kinetic Analysis

Kinematic data, kinetic data, and gait speed were calculated by the Visual 3D (C-motion Inc., Rockville, MD, United States) software. Kinematic variables were recorded for the left and right leg during the 6 m of level walking period for an average of three consecutive stride cycles. Motion and force data were used to define heel contact and toe-off for stride and step identification. Hip–knee–ankle angles in the sagittal plane were calculated between foot and shank, shank and thigh, and thigh and pelvis. The following parameters were analyzed: peak flexion and extension of the hip and knee joints; peak dorsiflexion and plantar flexion of the ankle joint; ROM of the ankle, knee, hip joints in the sagittal plane; and ankle, knee, and hip angles at the initial contact and toe-off. The gait speed was calculated simultaneously. Ankle, knee, and hip moments were calculated using the same software. Thereafter, peak moments of dorsiflexion and plantar flexion and peak flexion and extension moment of knee and hip were also analyzed.

### Statistical Analysis

The data were analyzed using SPSS (version 22.0, SPSS Inc., Chicago, IL, United States). Categorical variables were summarized using frequencies and percentages. Continuous variables were summarized using means and SDs. One-way analysis of variance (ANOVA) was applied to assess differences in characteristics between the three groups (MCI, SCD, and NC), while the chi-square test was used to assess differences in the distribution of gender, HIS, and comorbidities between the three groups. A *p*-value < 0.05 was considered statistically significant in these tests. As the gait kinematic and kinetic parameters are highly intercorrelated, a multivariate analysis of covariance (MANCOVA) was applied to detect the differences in gait kinematic and kinetic parameters on the left and right side across different groups with adjustment for age, gender, body mass index (BMI), gait speed, education years, diabetes mellitus, and GDS as covariates. A *p*-value < 0.002 (0.05/21) was considered statistically significant in MANCOVAs for 21 parameters (15 kinematic parameters and 6 kinetic parameters). Bonferroni corrected *post-hoc* analysis was used for multiple comparisons of 21 parameters across the three groups, and *p*-value < 0.0008 [0.05/(21 × 3)] was considered statistically significant (two-sided test). The results were reported as effect size and *p*-value.

## Results

**FIGURE 1:**
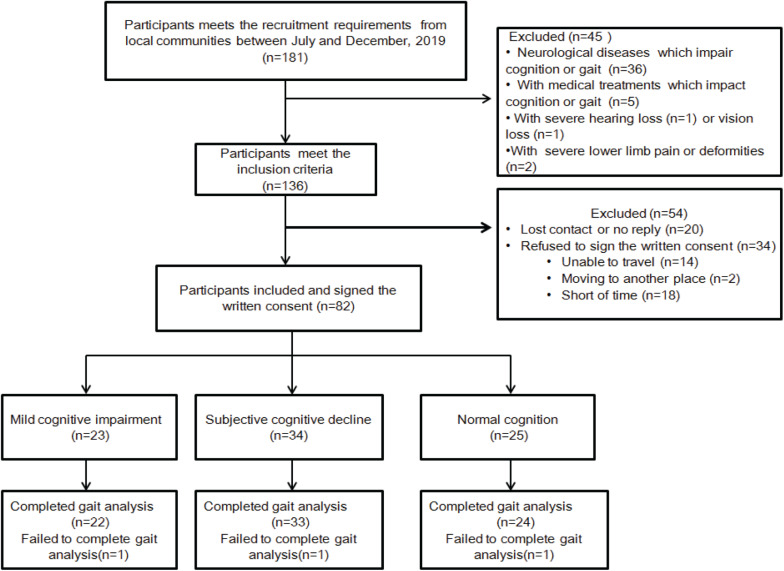
Flowchart of the recruitment.

The recruitment flowchart is shown in Figure 1. A total of 181 older adults were screened, of whom 136 met the inclusion criteria. A total of 82 men and women (50% each), aged 65–83 years, who signed the written consent were recruited for this study. However, 54 people were excluded due to loss of contact, no reply, or refusing to sign the written consent. Three individuals were not included in the data analysis because they failed to complete the gait analysis. Descriptive data of the participants’ cognitive impairment and demographic characteristics are presented in Table 1. Of the 79 individuals recruited for the study, 22 were diagnosed with MCI and 33 with SCD, leaving the remaining 24 with normal cognitive function. The mean age was 71.23 years (range, 61–84 years) for participants with MCI, 72.73 years (range, 59–82 years) for participants with SCD, and 72.73 years (range, 64–84 years) for participants with NC. Most of the participants had high school education (more than 12 years). Demographic characteristics and comorbidities were balanced among the three groups. Global cognition assessed using MMSE was statistically significant among the three groups (*p* = 0.004), showing a lower MMSE score for the MCI and SCD groups. In addition, the GDS score was also significantly different among the groups (*p* = 0.004), indicating higher depression in MCI and SCD participants. On the other hand, self-selected gait speed was not found to be different among these groups (*p* = 0.115).

**TABLE 1:** Baseline characteristics.

Characteristics	Total (*n* = 79)	MCI (*n* = 22)	SCD (*n* = 33)	NC (*n* = 24)	Group-wise comparison *F*(*p*-value)/χ^2^ (*p*-value)^*a*^
Age (years), mean (SD)	72.08 (5.65)	71.23 (6.65)	72.73 (5.25)	71.96 (5.30)	0.47 (0.629)
Female, *n* (%)	39 (49.37)	11 (50)	18 (54.55)	10 (41.67)	0.93 (0.629)^*a*^
Height (cm), mean (SD)	160.03 (8.34)	159.36 (6.19)	158.85 (8.12)	162.25 (10.09)	1.26 (0.290)
Weight (kg), mean (SD)	63.12 (10.67)	65.12 (10.31)	60.57 (9.02)	64.80 (12.64)	1.65 (0.198)
High blood pressure^+^, *n* (%)	34 (43.04)	8 (36.36)	15 (45.45)	11 (45.83)	0.56 (0.758)^*a*^
Diabetes, *n* (%)	15 (18.99)	3 (13.64)	8 (24.24)	4 (16.67)	1.09 (0.581)^*a*^
Lacunar infarction, *n* (%)	4 (5.06)	0 (0)	2 (6.06)	2 (8.33)	1.78 (0.412)^*a*^
**Hachinski Ischemia Scale**					9.39 (0.310)^*a*^
0, *n* (%)	35 (44.30)	13 (59.09)	11 (33.33)	11 (45.83)	
1, *n* (%)	26 (32.91)	3 (13.64)	15 (45.45)	8 (33.33)	
2, *n* (%)	11 (13.92)	4 (18.18)	5 (15.15)	2 (8.33)	
3, *n* (%)	6 (7.59)	2 (9.09)	2 (6.06)	2 (8.33)	
4, *n* (%)	1 (1.27)	0 (0)	0 (0)	1 (4.17)	
Education years, mean (SD)	12.66 (2.63)	11.73 (2.93)	13.15 (2.35)	12.83 (2.58)	2.07 (0.133)
MMSE, mean (SD)	27.35 (1.69)	26.91 (1.41)	26.97 (1.67)	28.29 (1.63)	**6.01 (0.004)**
GDS, mean (SD)	7.61 (5.12)	7.81 (5.46)	9.09 (4.84)	5.18 (4.49)	**4.19 (0.019)**
Gait speed (m/s), mean (SD)	1.06 (0.18)	1.01 (0.18)	1.11 (0.18)	1.04 (0.17)	2.23 (0.115)

*MCI, mild cognitive impairment; SCD, subjective cognitive decline; NC, normal cognition; SD, standard deviation; MMSE, Mini-Mental State Examination; GDS, Geriatric Depression Scale.*

*^+^High blood pressure is defined as systolic blood pressure >140 mmHg or diastolic blood pressure >90 mmHg.*

*^*a*^Chi-square test was applied. Values in bold are statistically significant, i.e., *p*-values < 0.05.*

### Gait Kinematic Characteristics of Participants With Mild Cognitive Impairment and Subjective Cognitive Decline

Multivariate analysis of covariance on the right side revealed that most of the independent variables were significantly related to gait performance (Wilk’s Lambda_*Group*_ = 0.29, *F* = 2.02, *p* = 0.004; Wilk’s Lambda_*Gender*_ = 0.62, *F* = 4.13, *p* < 0.001; Wilk’s Lambda_*BMI*_ = 0.37, *F* = 4.08, *p* < 0.001; Wilk’s Lambda_*Gait speed*_ = 0.16, *F* = 12.91, *p* < 0.001; Wilk’s Lambda_*Education years*_ = 0.46, *F* = 2.77, *p* = 0.003; Wilk’s Lambda_*DM*_ = 0.52, *F* = 2.22, *p* = 0.017) but not significant with age (Wilk’s Lambda_*Age*_ = 0.62, *F* = 1.45, *p* = 0.159) and GDS scale (Wilk’s Lambda_*GDS*_ = 0.70, *F* = 1.04, *p* = 0.437) (Table 2). However, on the left side, MANCOVA showed no significant multivariate effect of group on gait kinematics and kinetics (Table 2). Details about the kinematics and kinetics on the left and right sides are provided in Supplementary Table 1; the correlations between cognition and kinematics/kinetics are provided in Supplementary Table 2.

**TABLE 2:** Multivariate analysis of covariances of various covariates (left and right sides).

**Variable**	**Side**	**Effect**	**Value**	***F***	***p***	**Effect size (partial eta square)**
Group	Left	Wilk’s Lambda	0.50	0.98	0.521	0.30
	Right	Wilk’s Lambda	0.29	2.02	**0.004**	0.46
Age	Left	Wilk’s Lambda	0.58	1.69	0.080	0.42
	Right	Wilk’s Lambda	0.62	1.45	0.159	0.38
Gender	Left	Wilk’s Lambda	0.52	2.48	**0.008**	0.52
	Right	Wilk’s Lambda	0.37	4.13	**<0.001**	0.63
BMI	Left	Wilk’s Lambda	0.50	2.84	**0.004**	0.53
	Right	Wilk’s Lambda	0.37	4.08	**<0.001**	0.63
Gait speed	Left	Wilk’s Lambda	0.17	11.95	**<0.001**	0.83
	Right	Wilk’s Lambda	0.16	12.91	**<0.001**	0.84
Education years	Left	Wilk’s Lambda	0.63	1.38	0.194	0.37
	Right	Wilk’s Lambda	0.46	2.77	**0.003**	0.54
DM	Left	Wilk’s Lambda	0.67	1.13	0.362	0.33
	Right	Wilk’s Lambda	0.52	2.22	**0.017**	0.48
GDS	Left	Wilk’s Lambda	0.64	1.34	0.212	0.37
	Right	Wilk’s Lambda	0.70	1.04	0.437	0.30

*MANCOVA, multivariate analysis of covariance; BMI, body mass index; DM, diabetes mellitus; GDS, Geriatric Depression Scale.*

*Values in bold are statistically significant, i.e., *p*-values < 0.05.*

Multivariate analysis of covariances revealed a significant difference in knee peak extension angle (*F* = 8.77, *p* < 0.0001) and knee heel strike angle (*F* = 8.07, *p* = 0.001) among the three groups. However, no intergroup difference was observed for ankle and hip kinematic parameters in the sagittal plane. In addition, peak dorsiflexion, knee peak extension angle, knee ROM, and hip peak extension in the sagittal plane increased and hip heel strike angle and hip toe-off angle decreased with the progression of cognitive decline from normal to SCD and MCI.

### Gait Kinetic Characteristics

Gait kinetic parameters are shown in Table 3. MANCOVA analysis with age, BMI, and other factors as covariates did not show any intergroup differences (*p* > 0.002).

**TABLE 3:** Comparison of gait kinematic and kinetic parameters on the right side among the three groups (MANCOVA).

**Gait parameters**	**MCI (*n* = 19)**	**SCD (*n* = 30)**	**NC (*n* = 21)**	**MANCOVA *F* (*p-*value)^*a*^**	**Absolute difference**		
					**MCI vs. NC d (*p*-value)^*b*^**	**SCD vs. NC d (*p*-value)^*b*^**	**MCI vs. SCD d (*p*-value)^*b*^**
**Ankle kinematics in the sagittal plane, degree**							
Peak dorsiflexion (degree), mean (SD)	16.03 (3.20)	15.16 (3.28)	13.36 (3.24)	3.61 (0.033)	2.67 (0.033)	1.80 (0.203)	0.87 (1.000)
Peak plantar flexion (degree), mean (SD)	15.23 (4.78)	14.43 (4.89)	16.37 (4.83)	0.91 (0.407)	−1.14 (1.000)	−1.94 (0.547)	0.81 (1.000)
Ankle ROM (degree), mean (SD)	31.26 (4.30)	29.58 (4.40)	29.73 (4.34)	0.95 (0.393)	1.53 (0.803)	−0.15 (1.000)	1.68 (0.620)
Ankle heel strike angle (degree), mean (SD)	−2.11 (3.67)	−1.59 (3.75)	−4.08 (3.71)	2.74 (0.073)	1.98 (0.285)	2.49 (0.083)	−0.51 (1.000)
Ankle toe-off angle (degree), mean (SD)	0.84 (5.64)	−0.79 (5.77)	−0.48 (5.70)	0.48 (0.620)	1.31 (1.000)	−0.31 (1.000)	1.62 (1.000)
**Knee kinematics in the sagittal plane, degree**							
Knee peak flexion angle (degree), mean (SD)	65.17 (4.82)	67.33 (4.94)	65.79 (4.88)	1.18 (0.315)	−0.61 (1.000)	1.55 (1.000)	−2.16 (0.443)
Knee peak extension angle (degree), mean (SD)	−0.59 (4.54)	−4.60 (4.64)	−6.50 (4.58)	**8.77 (<0.0001)**	**5.91 (<0.0001)**	1.91 (0.503)	4.01 (0.016)
Knee ROM (degree), mean (SD)	64.58 (5.51)	62.74 (5.64)	59.28 (5.57)	4.77 (0.012)	5.30 (0.011)	3.46 (0.125)	1.84 (0.832)
Knee heel strike angle (degree), mean (SD)	5.22 (5.26)	10.46 (5.38)	11.43 (5.31)	**8.07 (0.001)**	−6.21 (0.001)^*c*^	−0.97 (1.000)	−5.24 (0.003)^*c*^
Knee toe-off angle (degree), mean (SD)	35.72 (5.85)	40.12 (5.98)	37.50 (5.91)	3.13 (0.051)	−1.79 (1.000)	2.62 (0.427)	−4.40 (0.049)
**Hip kinematics in the sagittal plane, degree**							
Hip peak flexion angle (degree), mean (SD)	36.72 (8.03)	40.64 (8.21)	41.37 (8.12)	1.94 (0.152)	−4.66 (0.218)	−0.73 (1.000)	−3.93 (0.343)
Hip peak extension angle (degree), mean (SD)	11.82 (7.90)	9.10 (8.09)	6.53 (7.99)	2.22 (0.117)	5.29 (0.118)	2.56 (0.858)	2.73 (0.787)
Hip ROM (degree), mean (SD)	48.54 (4.09)	49.74 (4.18)	47.91 (4.13)	1.15 (0.322)	0.63 (1.000)	1.83 (0.428)	−1.20 (1.000)
Hip heel strike angle (degree), mean (SD)	32.10 (7.92)	35.37 (8.10)	37.06 (8.00)	2.01 (0.143)	−4.96 (0.159)	−1.69 (1.000)	−3.27 (0.542)
Hip toe-off angle (degree), mean (SD)	−2.80 (8.16)	0.65 (8.35)	1.11 (8.24)	1.37 (0.262)	−3.90 (0.412)	−0.46 (1.000)	−3.46 (0.516)
**Gait kinetics in the sagittal plane**							
Peak dorsiflexion moment (N.m/kg), mean (SD)	0.08 (0.14)	0.03 (0.14)	0.02 (0.14)	1.22 (0.303)	0.07 (0.435)	0.01 (1.000)	0.05 (0.689)
Peak plantar flexion moment (N.m/kg), mean (SD)	1.60 (0.12)	1.58 (0.13)	1.55 (0.12)	0.65 (0.525)	0.05 (1.000)	0.02 (1.000)	0.02 (1.000)
Knee peak flexion moment (N.m/kg), mean (SD)	0.37 (0.15)	0.33 (0.16)	0.33 (0.16)	0.52 (0.600)	−0.04 (1.000)	0.01 (1.000)	0.05 (0.992)
Knee peak extension moment (N.m/kg), mean (SD)	0.50 (0.25)	0.54 (0.25)	0.57 (0.25)	0.50 (0.611)	−0.08 (0.974)	−0.03 (1.000)	−0.05 (1.000)
Hip peak flexion moment (N.m/kg), mean (SD)	0.63 (0.18)	0.58 (0.19)	0.61 (0.19)	0.37 (0.690)	0.02 (1.000)	−0.03 (1.000)	0.05 (1.000)
Hip peak extension moment (N.m/kg), mean (SD)	0.74 (0.26)	0.69 (0.27)	0.72 (0.27)	0.21 (0.808)	0.02 (1.000)	−0.03 (1.000)	0.05 (1.000)

*BMI, body mass index; GDS, Geriatric Depression Scale; MANCOVA, multivariate analysis of covariance; MCI, mild cognitive impairment; SCD, subjective cognitive decline; NC, normal cognition; SD, standard deviation; ROM, range of motion.*

*^*a*^MANCOVA with adjustment for age, BMI, gender, education years, diabetes mellitus, and gait speed as covariates, and the estimates of parameters with covariates evaluated at the following values: age = 71.93; BMI = 24.65; gender = 1.50 (male = 1, female = 2); education years = 12.70; diabetes mellitus = 1.20 (1 = without DM, 2 = with DM); gait speed = 1.06; GDS = 7.54. Values in bold are statistically significant, i.e., *p*-values < 0.002 for MANCOVAs. *F*-statistics has degree of freedom 2.67.*

*^*b*^Bonferroni correction was used in *post-hoc* comparisons across the three groups. Values in bold are statistically significant, i.e., *p*-values < 0.0008 for *post-hoc* tests.*

*^*c*^A trend of difference between groups (0.0008 < *p*-values < 0.01 for MANCOVA and *post-hoc* tests).*

### Results of *post-hoc* Analysis

**FIGURE 2:**
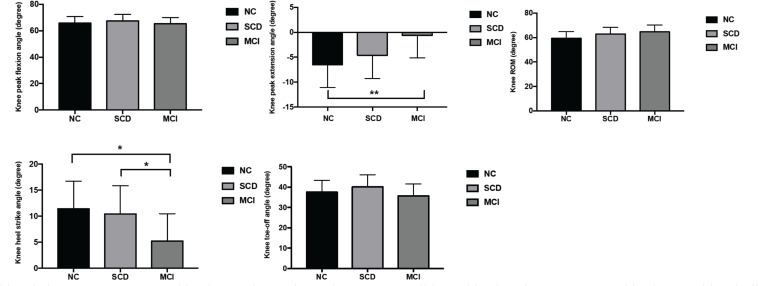
Comparison of right knee kinematics among the three groups (MANCOVA with Bonferroni *post-hoc* analysis). MANCOVA, multivariate analysis of covariance; MCI, mild cognitive impairment; SCD, subjective cognitive decline; NC, normal cognition; ROM, range of motion. *A trend of difference between groups (0.0008 < *p*-values < 0.01 for *post-hoc* tests). **p-Values < 0.0008 for *post-hoc* tests between MCI and NC groups.

Bonferroni *post-hoc* test was applied to explore the effect size and difference between MCI/NC, SCD/NC, and MCI/SCD (Table 3 and Figure 2). There was a significant difference of 5.91° [99.92, confidence interval (CI) 0.33–11.50, *p* < 0.0001] in knee peak extension in the MCI group compared with NC. Furthermore, there was a noticeable difference of −6.21° in knee heel strike angle (99.92% CI, -12.68–0.26, *p* = 0.001) and 5.30° increase in knee ROM (99.92% CI, −1.48–12.03, *p* = 0.011) in MCI compared with NC, as they were close to the level of significance. A trend of difference of −5.24° in knee heel strike angle (99.92% CI, −11.46–0.98, *p* = 0.005) was observed in MCI compared with SCD.

## Discussion

Although previous studies have found that older adults with MCI have slower gait speed and worse performance compared with healthy control, kinematic and kinetic changes in this population have not been reported. Our findings have shown that knee peak extension angle and knee angle at heel strike were significantly different among older adults with MCI, SCD, and NC. However, no intergroup differences in ankle and hip kinematics were found. In addition, knee peak extension angle was 5.91° larger while knee heel strike angle was 6.21° smaller in MCI compared with NC. These findings add more evidence that individuals with MCI have abnormal knee kinematics during level walking, which indicates that cognitive impairment may have a potential influence on gait kinematics along with changes in spatiotemporal parameters (Cohen et al., 2016).

Gait kinematic parameters, such as peak dorsiflexion, plantar flexion, ROM of ankle, peak flexion and extension, and ROM of knee and hip joint in the sagittal plane, are basic components of gait that influence postural control during walking. On the other hand, gait kinetic parameters, such as joint movement, power, and ground reaction force, indicate biomechanics of gait. Older adults show reduced ankle ROM, peak plantar flexion, increased knee and hip ROM, increased hip flexion angle at heel strike and peak hip flexion and peak hip extension and reduced dorsiflexion compared with young adults during walking (Boyer et al., 2017). In addition, reduced ankle strength and movement may lead to compensation of the knee and hip joint motion and power (Hortobágyi et al., 2016; Gueugnon et al., 2019). Furthermore, older adults with MCI and dementia have typical gait impairments, such as slower gait velocity, greater gait variability, and worse gait performance under dual-task conditions (Webster et al., 2006; Beauchet et al., 2014; Cohen et al., 2016; Rucco et al., 2016; Fuentes-Abolafio et al., 2020) and dual-task gait and slow gait speed, shorter step length, and high stride length variability are related to incident dementia (Ansai et al., 2017; Doi et al., 2019). While previous studies have focused on spatiotemporal gait parameters, the articular kinematic and kinetic characteristics of gait have not yet been reported in the older population with MCI or SCD. In this study, we have found a meaningful difference in knee peak extension and knee heel strike angle in the sagittal plane among MCI, SCD, and NC elderly. In addition, knee ROM was different among these groups. There was an increase in knee peak extension angle and knee ROM and a decrease in knee heel strike angle, indicating a more upright gait during the stance phase and a more flexed knee at heel strike in older adults with cognitive decline. This finding adds new insight into the gait characteristics of older adults with MCI and SCD.

Previous studies have reported the reduction in ankle ROM and plantar flexion in older adults (Boyer et al., 2017), which may be due to the weakness of ankle plantar flexors (Gueugnon et al., 2019). However, our results showed that although the difference in peak ankle dorsiflexion did not reach the adjusted statistically significant level among MCI, SCD, and NC, the values (2.67° and 1.80°) were clinically meaningful. Interestingly, one study about patients with Alzheimer’s disease (AD) and with behavior variant of frontotemporal dementia (bvFTD) also found a significant increase of dorsiflexion and a decrease of plantar flexion under cognitive dual-task conditions in AD patients compared to those of the normal control (Rucco et al., 2016). The observed increase in dorsiflexion may be due to weak planter flexors and overactive dorsiflexors in order to stabilize the ankle joint. Furthermore, we found an increase in knee peak extension angle and knee ROM during walking in MCI compared with healthy older adults, while previous studies found that healthy elderly showed an increased hip and knee ROM compared with young individuals (Boyer et al., 2017). This phenomenon may be due to weakness in ankle plantar flexors (Gueugnon et al., 2019) and biomechanical adjustment. Meanwhile, these findings indicate that cognitive decline and dementia might further aggravate abnormal posture in older adults during walking, leading to adaptive biomechanical changes for a stable posture. However, although we excluded participants with severe deformities of joints that affect walking, the ROM and muscle strength of the lower limbs were not recorded in our study. There might be slight differences in joint motions and muscle strength among the three groups, which may also contribute to the gait abnormalities. In addition, we did not find a significant difference in ankle and hip kinematics in the sagittal plane among older adults with MCI, SCD, and NC. Therefore, we conclude that ankle and hip kinematics may not be influenced to a noticeable extent in older people with cognitive decline under normal gait speed and single-task conditions.

The MCI participants in our study had complaints with memory loss, so they were considered in the early stage of AD. Therefore, their kinematic performances were not as significantly impaired as patients with dementia. Rucco et al. (2016) investigated the gait kinematics during single- and dual-task walking in patients with AD and bvFTD. They found that their articular kinematics were highly affected even during normal walking compared to healthy subjects. In the bvFTD group, impairment of the ankle, knee, and thigh in nearly all phases of the gait cycle was observed; however, in the AD group, impairment of knee and thigh kinematics was found. Furthermore, the gait performance of the AD group markedly deteriorated under dual-task conditions, which explains that the MCI participants in our study did not have entire impairment of all the ROMs under single-task conditions. Further research of walking under dual-task conditions is needed to explore the impairment of MCI participants.

*Post-hoc* analysis revealed increased knee peak extension and noticeable knee ROM and knee heel strike angle in MCI compared with NC, while no difference was found in SCD compared with NC. These findings indicated that knee kinematics were different significantly only in the MCI group; however, the SCD group had similar gait performance compared with NC. In addition, although the difference in ankle dorsiflexion did not reach the adjusted significant level, these values could be clinically important.

Previous studies have found that spatiotemporal parameters such as gait velocity, stride time, and stride length are correlated with cognitive domains of memory, executive function, and attention in the elderly with MCI (Xie et al., 2019). A recent study has shown that faster gait speed was associated with a change in immediate recall but not delayed recall memory (Sebastiani et al., 2020). While another study showed that executive function had a strong correlation with gait speed compared to other cognitive domains in patients with cognitive impairment (Toots et al., 2019). Although our findings indicate that older individuals with MCI have abnormal gait kinematics, no other study has reported the relationship between cognition and gait kinematics in individuals with MCI or SCD; therefore, further research is needed.

It has been observed that cognition and gait share the same anatomic substrates and brain control processes (Cohen et al., 2016). Gait impairment is typically associated with brain deterioration, especially gray matter atrophy and loss of white matter integrity (Ezzati et al., 2015; Wilson et al., 2019). In MCI patients, gait performance is correlated with gray matter volume, especially medial temporal (the hippocampus and parahippocampal gyrus) and left premotor cortex (Allali et al., 2016; McGough et al., 2018; Beauchet et al., 2019), white matter integrity (corpus callosum, forceps minor, and left inferior fronto-occipital fasciculus) (Snir et al., 2019), and reduced prefrontal activation during walking (Holtzer and Izzetoglu, 2020). Some studies have reported that executive function may impact gait performance in MCI patients (Xie et al., 2019) and is correlated with gait speed (Toots et al., 2019). However, gait parameters included in these studies were all spatiotemporal parameters and not kinematic or kinetic parameters. Therefore, although we observed an increase in knee extension angle and a decrease in heel strike angle in MCI participants, the relationship between gait kinematics and cognitive function needs further investigation. Furthermore, knee kinematics of MCI participants indicates that this population might have bad knee control during level walking. Therefore, clinical observation should not only evaluate cognitive performance of patients with MCI but also pay special attention to their gait characteristics along with other management strategies. Attention should be given to strength training of specific lower limb muscles such as knee extensors, flexors, as well as ankle plantar flexors to improve walking ability and balance control in these individuals.

Gait kinetics in our study population showed no meaningful difference among the three groups. This is the first study about gait kinetics in older people with MCI and SCD; therefore, negative findings may indicate that mild cognitive decline may not have a big impact on gait kinetics during level walking. Gait speed can influence joint moments and a stronger muscle contraction is required to produce high joint moments; therefore, older adults are less capable to produce a higher peak ankle moment when facing a higher task demand (Waanders et al., 2019). In this study, we chose self-selected walking speed during gait analysis, which might not be very challenging. A more difficult task such as dual-task walking or high-speed walking may be more sensitive to detect changes in joint moments among MCI, SCD, and cognitively normal older adults. Therefore, further studies are required to investigate the change in kinetic characteristics during different walking conditions and at various gait speeds.

Previous research has shown that age and gender play an important role in gait kinematics (Singhal et al., 2014; Boyer et al., 2017). In addition, obesity and high BMI have also been found to have a significant influence on gait kinematics, causing a large hip joint angle in both sagittal and transverse planes (Rosso et al., 2019) and a smaller hip ROM (Agostini et al., 2017). In addition, different walking speeds significantly influence gait kinetics (Waanders et al., 2019). Furthermore, diabetes mellitus (DM) was reported to have a potential influence on gait parameters simultaneously (Kimura et al., 2018). Therefore, we included all these factors as covariates in MANCOVA to avoid such an influence.

### Study Limitations

There are several limitations to the present study. First, no study has reported gait kinematic and kinetic characteristics in older adults with MCI and SCD, leading to an insufficiency in the discussion to compare our results to other findings. Second, our cross-sectional design could not reveal the causal influence of cognitive decline on gait kinematic and kinetic parameters in older adults; in this sense, a cohort study design would be much better to investigate the role of cognitive decline and gait characteristics. Finally, the sample size of the present study was not big enough to eliminate the influence of multiple factors on gait kinematic parameters, such as muscle weakness and limitation of joint movement.

## Conclusion

This study showed that an increase of right knee peak extension angle in the sagittal plane during level walking was found among older adults with MCI compared to those with NC. There was a noticeable increase in right knee ROM and a decrease of right knee heel strike angle in MCI participants compared to NC participants. It is also observed that gait kinetics was not significantly different among the three groups. This finding adds new evidence of gait abnormality in older adults with MCI. As recommendations for clinical practice, gait analysis should be thoroughly carried out to evaluate gait performance and knee joint angle should be particularly observed in older adults with MCI. Additionally, lower limb strength training should be advised to improve walking ability in these individuals. Furthermore, future research should include longitudinal studies with larger sample sizes to explore the impact of potential confounders on gait kinematics and kinetics and to reveal the brain’s structural and functional mechanism of gait kinematics in patients at early stages of dementia.

## Data Availability Statement

The raw data supporting the conclusions of this article will be made available by the authors, without undue reservation.

## Ethics Statement

The studies involving human participants were reviewed and approved by the Ethics Committee of The First Affiliated Hospital of Nanjing Medical University, Nanjing, China. The patients/participants provided their written informed consent to participate in this study.

## Author Contributions

YZ completed the funding application, managed and coordinated the study, and drafted the initial manuscript. TW provided the research ideas. QiaZ, YG, and XW applied for the ethical application, analyzed and collected gait data, and drafted the manuscript. HW and CS screened and diagnosed the participants, collected the data, and analyzed the characteristics of the participants. NA managed the experiment data. MX guided the study design and study process. QiuZ and YS did cognitive assessments and data analysis. LT and JM designed the study and did the statistical analysis. All the authors contributed to the article and approved the submitted version.

## Conflict of Interest

The authors declare that the research was conducted in the absence of any commercial or financial relationships that could be construed as a potential conflict of interest.

## Publisher’s Note

All claims expressed in this article are solely those of the authors and do not necessarily represent those of their affiliated organizations, or those of the publisher, the editors and the reviewers. Any product that may be evaluated in this article, or claim that may be made by its manufacturer, is not guaranteed or endorsed by the publisher.
