# Supplementary material for: Gait Kinematic and Kinetic Characteristics of Older Adults With Mild Cognitive Impairment and Subjective Cognitive Decline: A Cross-Sectional Study
Source: Front Aging Neurosci. 2021 Aug 3;13:664558. doi: 10.3389/fnagi.2021.664558 (PMC8368728; doi:10.3389/fnagi.2021.664558)
Supplement: Supplementary file 1 [file Table_1.docx]

Supplemental table 1 Intra-group and Inter-group comparisons of gait kinematics and kinetics of left and right side

| **Characteristics** |  | **Total**  **(n=79)** | **MCI**  **(n=22)** | **SCD**  **(n=33)** | **NC**  **(n=24)** | **Group-wise comparison**  ***F(P-*value)^a^** |
| --- | --- | --- | --- | --- | --- | --- |
| Ankle kinematics in sagittal plane, degree |  |  |  |  |  |  |
| Peak dorsiflexion (degree), mean (SD) | Left | 13.13(3.73) | **13.16(3.95)*** | **13.08(3.94)**** | 13.18(3.35) |  |
|  | Right | 15.00(3.29) | 15.75(3.42) | 15.71(3.12) | 13.33(2.90) |  |
|  | Left-right | -1.89(4.98) | -2.58(5.79) | -2.63(3.91) | -0.09(5.31) | 2.07(0.134) |
| Peak plantarflexion (degree), mean (SD) | Left | -15.06(3.07) | -14.92(3.54) | -15.37(3.35) | -14.72(4.81) |  |
|  | Right | -15.18(4.40) | -15.01(3.63) | -14.44(4.71) | -16.31(4.54) |  |
|  | Left-right | -0.06(5.22) | -0.31(5.32) | -1.16(4.90) | 1.59(5.33) | 1.97(0.146) |
| Ankle ROM (degree), mean (SD) | Left | 28.50(3.82) | **28.74(3.47)*** | 28.45(3.67) | 29.64(3.86) |  |
|  | Right | 30.12(4.08) | 30.75(3.79) | 30.04(4.46) | 29.22(3.98) |  |
|  | Left-right | -1.55(4.68) | -2.01(3.89) | -1.59(5.47) | -1.06(4.27) | 0.23(0.796) |
| Ankle heel strike angle (degree), mean (SD) | Left | 2.14(3.71) | 0.39(4.39) | **2.48(3.71)**** | **3.24(2.36)**** |  |
|  | Right | -2.68(3.80) | -2.88(4.06) | -1.90(3.57) | -3.57(3.78) |  |
|  | Left-right | 4.76(6.69) | 3.09(8.11) | 4.37(6.52) | 6.82(5.05) | 1.85(0.165) |
| Ankle toe-off angle (degree), mean (SD) | Left | -2.08(5.56) | -2.27(6.46) | -2.97(5.85) | -0.62(3.90) |  |
|  | Right | -0.24(5.20) | 0.29(5.71) | -0.36(5.28) | -0.55(4.81) |  |
|  | Left-right | -1.45(9.15) | -2.32(10.53) | -1.95(9.06) | 0.01(8.08) | 0.43(0.654) |
| Knee kinematics in sagittal plane, degree |  |  |  |  |  |  |
| Knee peak flexion angle (degree),  mean (SD) | Left | 67.19(6.41) | 67.93(6.70) | 66.26(6.32) | 67.78(6.36) |  |
|  | Right | 66.22(4.90) | 65.52(4.69) | 66.81(5.33) | 66.05 (4.55) |  |
|  | Left-right | 0.97(6.67) | 2.41(5.72) | -0.55(7.94) | 1.73(5.20) | 1.55(0.219) |
| Knee peak extension angle (degree),  mean (SD) | Left | -3.58(6.07) | -2.79(6.52) | -1.96(5.61) | -6.54(5.39) |  |
|  | Right | -4.14(5.60) | -1.68(5.52) | -3.77(4.16) | -6.91(6.35) |  |
|  | Left-right | 0.56(5.88) | -1.11(5.06) | 1.81(6.89) | 0.37(4.80) | 1.68(0.194) |
| Knee ROM (degree), mean (SD) | Left | 63.61(5.93) | 65.14(4.87) | 64.30(5.92) | **61.25(6.32)*** |  |
|  | Right | 62.08(5.99) | 63.84(5.81) | 63.04(5.68) | 59.14(5.71) |  |
|  | Left-right | 1.53(6.02) | 1.29(4.33) | 1.26(7.88) | 2.11(4.35) | 0.16(0.855) |
| Knee heel strike angle (degree), mean(SD) | Left | 9.99(5.31) | **8.82(5.67)*** | 9.75(5.37) | 11.45(4.72) |  |
|  | Right | 9.47(6.00) | 6.56(5.52) | 9.89(5.54) | 11.57(6.19) |  |
|  | Left-right | 0.58(3.40) | 2.27(4.71) | -0.14(4.63) | -0.19(4.79) | 2.11(0.129) |
| Knee toe-off angle (degree),mean(SD) | Left | 36.14(10.18) | 35.02(9.74) | **34.29(11.44)*** | 39.86(7.79) |  |
|  | Right | 38.01(6.02) | 36.90(4.43) | 38.31(6.85) | 38.60(6.15) |  |
|  | Left-right | -1.45(9.15) | -1.88(9.09) | -4.03(9.73) | 1.30(5.15) | 2.70(0.074) |
| Hip kinematics in sagittal plane, degree |  |  |  |  |  |  |
| Hip peak flexion angle (degree), mean (SD) | Left | 40.24(9.18) | 39.78(9.33) | 39.13(10.72) | 42.20(6.33) |  |
|  | Right | 39.44(8.67) | 38.14(8.59) | 38.95(9.67) | 41.32(7.21) |  |
|  | Left-right | 0.80(9.99) | 1.64(4.21) | 0.18(14.65) | 0.89(4.77) | 0.14(0.871) |
| Hip peak extension angle (degree),  mean (SD) | Left | 9.60(10.57) | 8.84(9.84) | 13.18(10.58) | 5.36(9.84) |  |
|  | Right | 9.11(10.47) | 9.17(9.64) | 11.96(10.12) | 5.13(10.78) |  |
|  | Left-right | 0.49(9.74) | -0.33(3.71) | 1.22(14.38) | 0.23(4.46) | 0.18(0.839) |
| Hip ROM (degree), mean (SD) | Left | 49.84(5.53) | 48.62(4.48) | 53.30(5.52) | 47.57(5.27) |  |
|  | Right | 48.55(5.59) | 47.31(4.61) | 50.91(4.88) | 46.45(6.26) |  |
|  | Left-right | 1.29(5.26) | 1.30(3.75) | 1.40(7.04) | 1.12(3.49) | 0.02 (0.982) |
| Hip heel strike angle (degree), mean (SD) | Left | 35.21(8.39) | **34.87(8.68)*** | 34.05(9.39) | 37.216(6.31) |  |
|  | Right | 34.67(8.21) | 33.15(8.50) | 33.99(8.49) | 37.01(7.31) |  |
|  | Left-right | 0.58(3.40) | 1.72(3.38) | 0.07(3.02) | 0.23(3.82) | 1.75(0.180) |
| Hip toe-off angle (degree), mean (SD) | Left | -0.89(11.70) | -0.34(10.82) | **-4.56(11.96)*** | 3.84(10.66) |  |
|  | Right | -0.36(10.57) | -0.10(9.90) | -2.73(10.88) | 2.67(10.34) |  |
|  | Left-right | -0.46(4.63) | -0.24(5.04) | -1.83(4.03) | 1.30(4.58) | 3.33(0.041) |
| Gait kinetics in sagittal plane |  |  |  |  |  |  |
| Peak dorsiflexion moment (N.m/Kg),  mean(SD) | Left | 0.04(0.05) | 0.04(0.06) | 0.05(0.06) | 0.02(0.02) |  |
|  | Right | 0.04(0.14) | 0.08(0.26) | 0.03(0.03) | 0.01(0.02) |  |
|  | Left-right | -0.00(0.12) | -0.04(0.21) | 0.02(0.06) | 0.00(0.01) | 2.02(0.141) |
| Peak plantar flexion moment (N.m/Kg),  mean(SD) | Left | 1.54(0.17) | 1.52(0.16) | 1.57(0.18) | 1.52(0.17) |  |
|  | Right | 1.57(0.19) | 1.54(0.16) | 1.60(0.19) | 1.55(0.21) |  |
|  | Left-right | -0.02(0.11) | -0.02(0.08) | -0.02(0.14) | -0.03(0.10) | 0.02(0.976) |
| Knee peak flexion moment (N.m/Kg),  mean(SD) | Left | 0.36(0.17) | 0.33(0.14) | 0.38(0.19) | 0.35(0.16) |  |
|  | Right | 0.34(0.17) | 0.33(0.16) | 0.34(0.18) | 0.34(0.17) |  |
|  | Left-right | 0.02(0.11) | 0.00(0.10) | 0.04(0.13) | 0.01(0.11) | 0.96(0.388) |
| Knee peak extension moment (N.m/Kg),  mean(SD) | Left | 0.56(0.24) | 0.45(0.23) | 0.61(0.23) | 0.57(0.24) |  |
|  | Right | 0.52(0.25) | 0.43(0.22) | 0.58(0.23) | 0.54(0.28) |  |
|  | Left-right | 0.03(0.22) | 0.03(0.19) | 0.04(0.22) | 0.02(0.27) | 0.06(0.939) |
| Hip peak flexion moment (N.m/Kg),  mean(SD) | Left | 0.58(0.23) | 0.55(0.22) | 0.62(0.23) | 0.54(0.22) |  |
|  | Right | 0.58(0.23) | 0.53(0.21) | 0.62(0.23) | 0.57(0.24) |  |
|  | Left-right | -0.01(0.14) | 0.02(0.21) | -0.00(0.11) | -0.04(0.09) | 0.77(0.466) |
| Hip peak extension moment (N.m/Kg),  mean (SD) | Left | 0.73(0.30) | 0.68(0.25) | 0.75(0.35) | 0.74(0.28) |  |
|  | Right | 0.70(0.29) | 0.66(0.28) | 0.71(0.31) | 0.72(0.28) |  |
|  | Left-right | 0.03(0.21) | 0.02(0.16) | 0.05(0.23) | 0.01(0.22) | 0.25(0.779) |

Abbreviation: MCI=mild cognitive impairment, SCD= subjective cognitive decline, NC=normal cognition, SD = standard deviation.

Note: Significant difference are marked in the table by * and ** according to the paired sample t- test in comparison of kinematics and kinetics between left and right side.

* = significant at the *P* = 0.05 level , ** =significant at the *P* = 0.01 level.

a One-way ANOVA was applied to detect inter-group difference. The adjusted significant level is P value <0.017.

Supplemental table 2 Correlations between cognitive assessments and kinematics/kinetics

|  | **Memory** | | **Executive** | | **Language** |  | **Global cognition** |
| --- | --- | --- | --- | --- | --- | --- | --- |
|  | **Short time recall**  **( AVLT-H)**  ***r*(*P*)** | **Delayed Recall**  **(AVLT-H)**  ***r*(*P*)** | **TMT-A**  ***r*(*P*)** | **TMT-B**  ***r*(*P*)** | **BNT**  ***r*(*P*)** | **VFT**  ***r*(*P*)** | **MMSE**  ***r*(*P*)** |
| Ankle kinematics in sagittal plane, degree |  |  |  |  |  |  |  |
| Peak dorsiflexion (degree) | -0.57(0.670) | **-0.26(0.044)*** | 0.10(0.457) | -0.07(0.600) | 0.18(0.183) | -0.21(0.113) | -0.16(0.222) |
| Peak plantarflexion (degree) | -0.06(0.676) | 0.04(0.787) | 0.18(0.179) | 0.01(0.963) | 0.01(0.952) | -0.120.350) | -0.25(0.053) |
| Ankle ROM (degree) | 0.02(0.881) | -0.246(0.060) | -0.13(0.336) | -0.06(0.644) | 0.128(0.335) | -0.02(0.882) | 0.17(0.209) |
| Ankle heel strike angle (degree) | -0.03(0.820) | -0.14(0.300) | 0.03(0.838) | -0.12(0.381) | 0.08(0.540) | -0.25(0.053) | 0.09(0.515) |
| Ankle toe-off angle (degree) | 0.01(0.967) | -0.11(0.388) | 0.04(0.757) | -0.05(0.710) | **0.26(0.049)*** | -0.02(0.912) | -0.17(0.189) |
| Knee kinematics in sagittal plane, degree |  |  |  |  |  |  |  |
| Knee peak flexion angle (degree) | **0.38(0.003)**** | -0.03(0.831) | 0.08(0.561) | -0.05(0.731) | 0.11(0.389) | 0.01(0.986) | 0.20(0.131) |
| Knee peak extension angle (degree) | -0.24(0.064) | **-0.26(0.049)*** | 0.00(0.983) | -0.15(0.266) | -0.01(0.922) | -0.25(0.059) | -0.05(0.731) |
| Knee ROM (degree) | 0.11(0.410) | -0.24(0.070) | 0.07(0.617) | 0.08(0.224) | -0.20(0.126) | 0.11(0.391) | **0.33(0.011)*** |
| Knee heel strike angle (degree) | **0.37(0.004)**** | 0.20(0.139) | -0.03(0.839) | 0.07(0.582) | 0.14(0.308) | **0.28(0.032)*** | 0.01(0.966) |
| Knee toe-off angle (degree) | 0.16(0.216) | -0.05(0.716) | 0.06(0.650) | **-**0.22(0.098) | -0.00(0.976) | 0.07(0.596) | 0.06(0.634) |
| Hip kinematics in sagittal plane, degree |  |  |  |  |  |  |  |
| Hip peak flexion angle (degree) | **0.26(0.047)*** | -0.09(0.488) | -0.04(0.779) | -0.04(0.758) | -0.00(0.974) | 0.04(0.745) | 0.17(0.212) |
| Hip peak extension angle (degree) | -0.15(0.247) | 0.03(0.828) | 0.03(0.822) | -0.06(0.678) | 0.07(0.584) | -0.03(0.829) | -0.06(0.642) |
| Hip ROM (degree) | 0.22(0.094) | -0.13(0.334) | -0.02(0.905) | -0.19(0.146) | 0.05(0.701) | -0.07(0.621) | 0.05(0.713) |
| Hip heel strike angle (degree) | 0.24(0.068) | -0.06(0.65) | -0.05(0.690) | 0.02(0.909) | 0.02(0.855) | 0.04(0.780) | 0.02(0.912) |
| Hip toe-off angle (degree) | 0.14(0.295) | -0.10(0.470) | 0.00(0.982) | 0.03(0.819) | -0.19(0.158) | -0.03(0.815) | 0.02(0.885) |
| Gait kinetics in sagittal plane |  |  |  |  |  |  |  |
| Peak dorsiflexion moment(N.m/Kg ), mean(SD) | -0.21(0.113) | -0.18(0.182) | 0.03(0.802) | 0.12(0.377) | 0.16(0.230) | -0.05(0.708) | -0.11(0.403) |
| Peak plantar flexion moment (N.m/Kg), mean(SD) | 0.19(0.148) | -0.08(0.547) | -0.10(0.475) | -0.04(0.744) | 0.027(0.840) | -0.08(0.559) | -0.03(0.803) |
| Knee peak flexion moment (N.m/Kg), mean(SD) | 0.13(0.311) | 0.11(0.412) | -0.16(0.240) | 0.00(0.999) | -0.09(0.478) | -0.10(0.457) | 0.04(0.748) |
| Knee peak extension moment (N.m/Kg),mean(SD) | 0.15(0.270) | -0.00(0.976) | 0.05(0.737) | 0.17(0.737) | 0.03(0.843) | 0.09(0.495) | -0.08(0.537) |
| Hip peak flexion moment (N.m/Kg), mean(SD) | -0.05(0.704) | -0.02(0.891) | -0.11(0.395) | -0.19(0.150) | -0.19(0.148) | 0.11(0.394) | -0.07(0.627) |
| Hip peak extension moment (N.m/Kg), mean (SD) | 0.04(0.786) | 0.12(0.360) | -0.22(0.098) | -0.15(0.269) | -0.04(0.758) | -0.12(0.380) | 0.05(0.684) |

Abbreviation:

Note: Significant difference are marked in the table by * and ** according to the partial correlation test controlling for gender, BMI, gait speed, education years and DM.

* = significant at the *P* = 0.05 level , ** =significant at the *P* = 0.01 level.
